# Neuromyelitis optica spectrum disorders without and with autoimmune diseases

**DOI:** 10.1186/s12883-014-0162-7

**Published:** 2014-08-19

**Authors:** Bingjun Zhang, Yi Zhong, Yanqiang Wang, Yongqiang Dai, Wei Qiu, Lei Zhang, Haiyan Li, Zhengqi Lu

**Affiliations:** 1 Department of Neurology, The Third Affiliated Hospital of Sun Yat-sen University, No 600 Tianhe Road, Guangzhou 510630, Guangdong, China; 2 Department of Rheumatology and Immunology, The Third Affiliated Hospital of Sun Yat-sen University, Guangzhou, China

**Keywords:** Neuromyelitis optica, Neuromyelitis optica spectrum disorder, Non-organ-specific autoimmune diseases, Organ-specific autoimmune diseases, Autoantibodies, Magnetic resonance imaging

## Abstract

**Background:**

Neuromyelitis optica spectrum disorder (NMOSD) can coexist with non-organ-specific or organ-specific autoimmune diseases. The aim of this study was to investigate and compare the features between NMOSD without and with autoimmune diseases, and NMOSD with non-organ-specific and organ-specific autoimmune diseases.

**Methods:**

One hundred and fifty five NMOSD patients without autoimmune diseases (n = 115) and with autoimmune diseases (n = 40) were enrolled. NMOSD with autoimmune diseases were divided by organ-specific autoimmune diseases. The clinical, laboratory and magnetic resonance imaging features between two groups were assessed.

**Results:**

Motor deficit was less frequent in NMOSD patients with non-organ-specific autoimmune diseases (p = 0.024). Cerebrospinal fluid white blood cell and protein, serum C-reactive protein and immunoglobulin G were lower in NMOSD patients without autoimmune diseases, while several autoantibodies seropositivity and thyroid indexes were significantly higher in NMOSD patients with autoimmune diseases (p < 0.05). No difference was found in other clinical and laboratory characteristics between different NMOSD subtypes (p > 0.05). NMOSD patients with autoimmune diseases had higher brain abnormalities than NMOSD without autoimmune diseases (p < 0.001).

**Conclusions:**

The characteristics between NMOSD without and with autoimmune diseases were similar. NMOSD with autoimmune diseases have high frequency of brain abnormalities.

## Background

Neuromyelitis optica (NMO) is a severe demyelinating disease of the central nervous system that affects the optic nerve and spinal cord but has protean and diverse potential clinical and radiological manifestations [1]-[3]. The broadened array of disorders associated with NMO immunoglobulin G (IgG) has been termed ‘NMO spectrum disorders’ (NMOSD), the diagnosis of which is greatly facilitated by the association of NMOSD with a specific biomarker for NMO, NMO-IgG [1]. Several groups have recognized a strong association of NMOSD with non-organ-specific autoimmune diseases (e.g. systemic lupus erythematosus (SLE), Sjögren syndrome (SS), rheumatoid arthritis (RA), undifferentiated connective tissue disease (UCTD)), and organ-specific autoimmune diseases (e.g. thyroid diseases, myasthenia gravis) [4]-[6]. However, few systemic studies have focused on the relationship between NMOSD without and with autoimmune diseases, and NMOSD with non-organ-specific and organ-specific autoimmune diseases. The characteristics of different NMOSDs, particularly NMOSD with non-organ-specific and organ-specific autoimmune diseases, were not studied enough. In this study, we investigated and compared the clinical, laboratory and magnetic resonance imaging (MRI) features between NMOSD without and with autoimmune diseases. Furthermore, the characteristics of NMOSD with non-organ-specific and organ-specific autoimmune diseases were also investigated.

## Methods

### Patients

Our database comprised 170 Chinese patients with NMOSD who were diagnosed and admitted from March 1, 2002 to March 1, 2013 in the MS Center of the Third Affiliated Hospital of Sun Yat-sen University, Guangzhou, China. NMO was diagnosed according to the 2006 Wingerchuk criteria [2]. In addition, NMOSD was diagnosed according to the 2007 Wingerchuk criteria [1]. Other included were: (a) all of these patients whose serum samples were tested for NMO-IgG, autoreactive antibodies (antinuclear antibodies (ANAs), extractable nuclear antigen autoantibodies (ENAs), rheumatoid factors (RFs) anti-neutrophil cytoplasmic antibodies (ANCAs)), immunoglobulins, complements, thyroid hormones and autoantibodies; and (b) also MRI of the brain and spinal cord available for review. Non-organ-specific autoimmune diseases (e.g. SLE [7], SS [8], RA [9], UCTD [10]), and organ-specific autoimmune diseases (e.g. thyroid diseases) were diagnosed by neurologists/rheumatologists/endocrinologists according to the criteria and typology guidelines. Clinical data and MRI scans were collected from these individuals, a group that including 115 NMOSD patients without autoimmune diseases and 40 with autoimmune diseases (20 with non-organ-specific autoimmune diseases and 18 with organ-specific autoimmune diseases).

This study was approved by the local Ethics Committee of the Third Affiliated Hospital of Sun Yat-sen University. Informed consents for this investigation were obtained from the patients or their family members.

### Laboratory testing

Blood and cerebrospinal fluid (CSF) samples were obtained from all the patients in our study during hospital admission. All profiling for each patient was performed using commercially clinical laboratory assays. CSF oligoclonal banding (OCB) and NMO-IgG were tested in our clinical neuroimmunological Laboratory. Autoreactive antibodies (ANAs, ENAs, RFs, ANCAs) testing were performed at the clinical rheumatology immunology laboratory of Sun Yat-sen University. The immunoglobulins, complements, thyroid indexes, and other profiling were tested in the clinical laboratory of Sun Yat-sen University.

### Magnetic resonance imaging

Brain and spinal cord MRI scans were performed in all patients using a GE 1.5 T MR scanner (General Electric, Milwaukee, Wisconsin, USA). The slice thickness of the axial scans was 5 mm. Conventional MRI protocols were used in all patients: T1 with and without gadolinium enhancement (400/15.5 ms, TR/TE) and T2 (2500– 3500/100 ms, TR/TE) for spinal cord MRI; and T1 with and without gadolinium enhancement (2128–2300/11.6–12.4 ms, TR/TE), T2 (4600–4640/97.8–102 ms, TR/TE), and fluid-attenuated inversion recovery (FLAIR) (8800/120 ms, TR/TE) for brain MRI. Each patient underwent MRI scanning at the time of the initial diagnosis, prior to corticosteroid treatment. No patients were receiving immunomodulatory treatment at the time of the MRI scanning. The numbers, locations, and diameters of lessions were recorded. All image archives were reviewed with a DICOM viewer on a Macintosh computer. An experienced neuroradiologist and a neurologist, both of whom were blinded to the diagnostic categorization and the patients’ clinical features, each analyzed all of the MRI scans. The final assessments were made by consensus.

### Statistical analysis

Statistical analysis was performed by SPSS version 22.0. Values of p = 0.05 were considered statistically significant. Quantitative data were processed using the Mann–Whitney U-test or Student’s t-test. All quantitative data in this study are presented as mean ± standard deviation (SD) or median (range). Qualitative data were analyzed with the χ^2^ test or Fisher’s exact test. Power calculations indicated these were 80% power to detect 9.7% (n = 155) and 27.6% (n = 40) differences in the percentage of features, with alpha set at 0.05.

## Results

**Figure 1:**
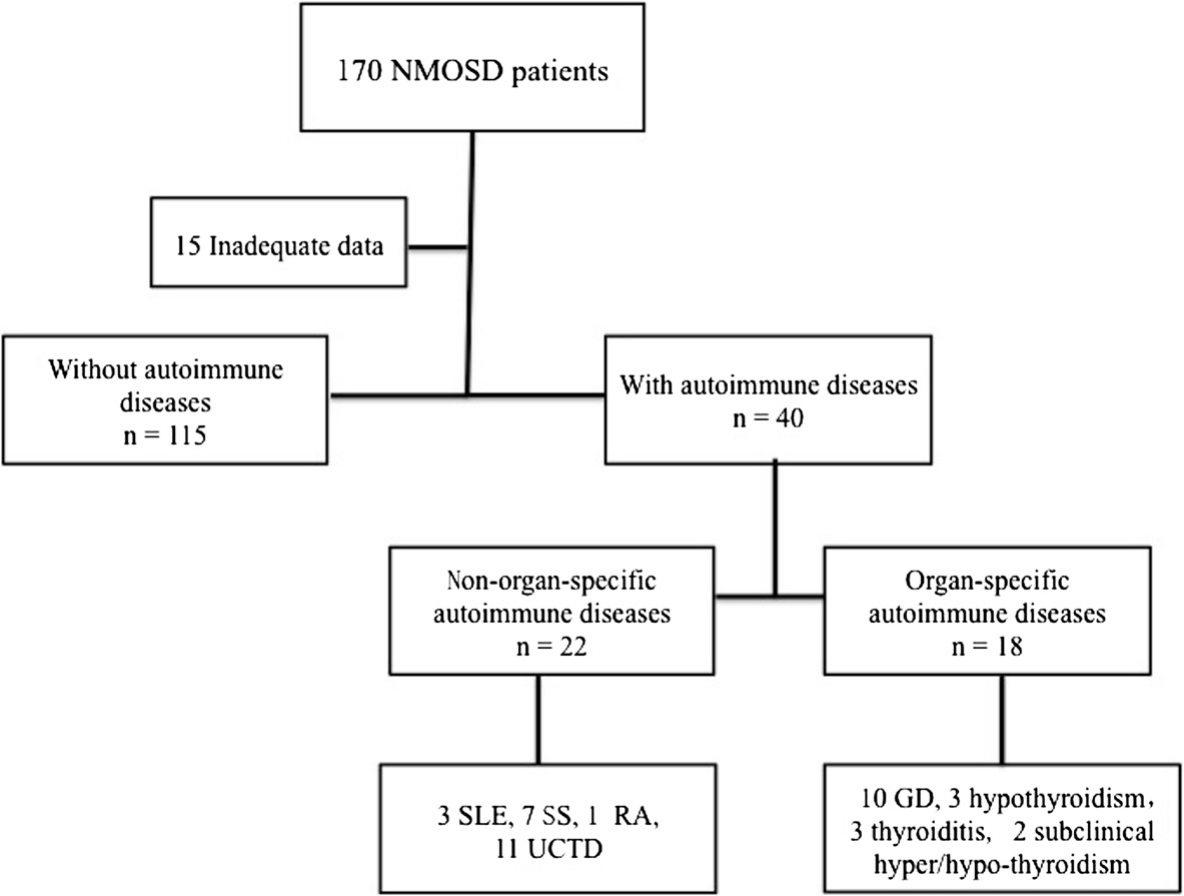
**Study flowchart.** Abbreviations: SLE = systemic lupus erythematosus; SS = Sjögren syndrome; RA = rheumatoid arthritis; UCTD = undifferentiated connective tissue disease; GD = Graves’ disease.

The data of 170 patients with NMOSD were reviewed between 2002 and 2013. A total of 155 patients satisfied the diagnostic criteria for inclusion in this study: 115 NMOSD patients without autoimmune diseases and 40 with autoimmune diseases (22 NMOSD patients with non-organ-specific autoimmune diseases and 18 NMOSD patients with organ-specific autoimmune diseases). The details of the enrollment process can be seen in the flowchart (Figure 1).

The demographic and clinical features of the patients are summarized in Table 1. There were no statistical differences in demographic and clinical characteristics between NMOSD without and with autoimmune diseases (p > 0.05). After further analysis, motor deficit was less frequent in NMOSD patients with non-organ-specific autoimmune diseases than in NMOSD patients with organ-specific autoimmune diseases (p = 0.024). No difference was found in other demographic and clinical features between NMOSD patients with non-organ-specific autoimmune diseases and NMOSD patients with organ-specific autoimmune diseases (p > 0.05).

**Table 1:** Demographic and clinical characteristics between NMOSD without and with autoimmune diseases, and between NMOSD with non-organ-specific and organ-specific autoimmune diseases

	**NMOSD**			**NMOSD with autoimmune diseases**		
	**Without autoimmune diseases (n = 115)**	**With autoimmune diseases (n = 40)**	**P**	**Non-organ-specific (n = 22)**	**Organ-specific (n = 18)**	**P**
Gender, F: M	91: 24	37: 3	0.055	22: 0	15: 3	0.165
Age, years	37.63 ± 14.33	40.71 ± 13.80	0.240	40.90 ± 14.86	40.47 ± 12.80	0.924
Age at onset, years	34.20 ± 13.70	37.37 ± 13.50	0.207	36.91 ± 14.63	37.94 ± 12.37	0.814
Disease duration, years	1.83 (0.08, 30.00)	1.33 (0.08, 31.08)	0.219	1.25 (0.08, 31.08)	2.67 (0.08, 7.00)	0.414
Annualized relapse rate	0.66 (0, 12.00)	0.89 (0, 6.00)	0.183	0.80 (0, 3.43)	0.95 (0, 6.00)	0.784
EDSS at last visit	2.0 (0, 23.0)	2.5 (0, 10.0)	0.067	2.5 (1.0, 8.5)	3.0 (0, 10.0)	0.529
Clinical features, n (%)						
Headache	18 (15.7%)	8 (20.0%)	0.526	3 (13.6%)	5 (27.8%)	0.475
Nystagmus	6 (5.2%)	4 (10.0%)	0.289	2 (9.1%)	2 (11.1%)	0.832
IHN	26 (22.6%)	12 (30.0%)	0.349	4 (18.2%)	8 (44.4%)	0.071
Bulbar paralysis	9 (7.8%)	2 (5.0%)	0.549	1 (4.5%)	1 (5.6%)	1.000
Bowel or bladder dysfunction	49 (42.6%)	18 (45.0%)	0.793	7 (31.8%)	11 (61.1%)	0.064
Visual impairment	98 (85.2%)	33 (82.5%)	0.682	18 (81.8%)	15 (83.3%)	0.900
Motor deficit	68 (59.1%)	21 (52.5%)	0.465	8 (36.4%)	13 (72.2%)	0.024*
Sensory deficit	83 (72.2%)	25 (62.5%)	0.252	14 (63.6%)	11 (61.1%)	0.870
Neuropathic pain	29 (25.4%)	13 (32.5%)	0.388	6 (27.3%)	7 (38.9%)	0.435

*Abbreviations: EDSS* Expanded Disability Status Scale, *F: M* female: male, *IHN* intractable hiccup and nausea, *NMOSD* neuromyelitis optica spectrum disorders.

*p < 0.05.

The laboratory features of the patients are summarized in Table 2. CSF white blood cell (WBC) and protein were significantly lower in NMOSD patients without autoimmune diseases than in NMOSD patients with autoimmune diseases (p < 0.05), while CSF glucose was significantly higher in NMOSD patients without autoimmune diseases than in NMOSD patients with autoimmune diseases (p = 0.012). Serum C-reactive protein (CRP) was significantly lower in NMOSD patients without autoimmune diseases than NMOSD patients with autoimmune diseases (p = 0.017). Furthermore, ANA, anti-SSA/Ro antibodies (SSA), anti-SSB/La antibodies (SSB), anti-double stranded DNA antibodies (Ds-DNA), anti-nucleosome antibody (AnuA), anti-Sm antibodies (Sm) seropositivity, and serum IgG were significantly lower in NMOSD patients without autoimmune diseases than in NMOSD patients with autoimmune diseases (p < 0.05), while anti-thyroglobulin antibodies (TG) seropositivity, thyroxin (T4), and free thyroxin (FT4) were significantly higher in NMOSD patients with autoimmune diseases (p < 0.05). NMO-IgG was detectable in 67.0% (71/115) NMOSD without autoimmune diseases and in 80.0% (32/40) NMOSD patients with autoimmune diseases (p = 0.120).

**Table 2:** Laboratory features between NMOSD without and with autoimmune diseases, and between NMOSD with non-organ-specific and organ-specific autoimmune diseases

	**NMOSD**			**NMOSD with autoimmune diseases**		
	**Without autoimmune diseases (n = 115)**	**With autoimmune diseases (n = 40)**	**P**	**Non-organ-specific (n = 22)**	**Organ-specific (n = 18)**	**P**
**CSF Index**						
WBC (10^6^)	6 (0, 140)	10 (0, 48)	0.002*	12 (2, 48)	9.5 (0, 46)	0.307
Protein (g/l)	0.22 (0.06, 1.64)	0.27 (0.12, 0.80)	0.017*	0.27 (0.19, 0.80)	0.32 (0.12, 0.72)	0.754
Glucose (mmol/l)	3.47 ± 0.70	3.11 ± 0.94	0.012*	2.88 ± 0.23	3.41 ± 1.59	0.075
Chloride (mmol/l)	126.3 ± 5.1	126.6 ± 2.8	0.657	126.3 ± 6.9	127.0 ± 9.7	0.434
OCB, n (%)	6 (5.2%)	3 (7.5%)	0.595	2 (9.1%)	1 (5.6%)	1.000
**Serums Index**						
CRP (mg/l)	2.33 ± 4.37	5.63 ± 7.62	0.001*	2.16 (0.00, 26.09)	0.80 (0.10, 15.00)	0.289
ESR (mm/H)	12.90 ± 7.89	14.04 ± 13.39	0.519	10.5 (1.3, 50.0)	7.00 (3.0, 24.0)	0.414
NMO-IgG, n (%)	71 (67.0%)	32 (80.0%)	0.120	18 (81.8%)	14 (77.8%)	0.751
ANA, n (%)	16 (13.9%)	20 (50.0%)	<0.001**	18 (81.8%)	2 (11.1%)	<0.001**
SSA, n (%)	2 (1.7%)	20 (50.0%)	<0.001**	20 (90.9%)	0 (00.0%)	<0.001**
SSB, n (%)	1 (0.9%)	11 (27.5%)	<0.001**	11 (50.0%)	0 (00.0%)	0.002*
RF, n (%)	2 (1.7%)	3 (7.5%)	0.075	2 (9.10%)	1 (5.60%)	1.000
Ds-DNA, n (%)	0 (0.0%)	5 (12.5%)	0.001*	5 (22.7%)	0 (00.0%)	0.053
AnuA, n (%)	0 (0.0%)	3 (7.5%)	0.021*	3 (13.6%)	0 (00.0%)	0.305
AHA, n (%)	0 (0.0%)	1 (2.5%)	0.579	1 (4.5%)	0 (00.0%)	1.000
RNP, n (%)	0 (0.0%)	2 (5.0%)	0.110	2 (9.1%)	0 (00.0%)	0.492
Sm, n (%)	0 (0.0%)	3 (7.5%)	0.021*	3 (13.6%)	0 (00.0%)	0.305
Jo-1, n (%)	0 (00.0%)	0 (00.0%)	-	0 (00.0%)	0 (00.0%)	-
Scl-70, n (%)	0 (00.0%)	0 (00.0%)	-	0 (00.0%)	0 (00.0%)	-
ANCA, n (%)	0 (00.0%)	0 (00.0%)	-	0 (00.0%)	0 (00.0%)	-
Rib-P, n (%)	0 (0.0%)	2 (5.0%)	0.110	2 (9.1%)	0 (00.0%)	0.492
AKA, n (%)	0 (00.0%)	0 (00.0%)	-	0 (00.0%)	0 (00.0%)	-
RA33, n (%)	0 (00.0%)	0 (00.0%)	-	0 (00.0%)	0 (00.0%)	-
CCP, n (%)	0 (00.0%)	0 (00.0%)	-	0 (00.0%)	0 (00.0%)	-
IgG (g/l)	12.46 ± 4.95	18.33 ± 0.82	<0.001**	14.18 (7.00, 38.80)	12.61 (7.00, 33.22)	0.765
IgA (g/l)	1.41 ± 0.40	1.51 ± 4.95	0.310	1.30 (0.45, 3.60)	1.28 (0.56, 2.15)	0.142
IgM (g/l)	1.15 ± 0.37	1.06 ± 0.38	0.169	1.14 (0.45, 1.75)	0.97 (0.58, 1.99)	0.454
C3 (g/l)	1.09 ± 0.24	1.04 ± 0.39	0.334	1.06 ± 0.17	1.01 ± 0.13	0.667
C4 (g/l)	0.23 ± 0.15	0.19 ± 0.15	0.116	0.15 (0.04, 0.93)	0.15 (0.03, 0.32)	0.935
CH50 (U/ml)	44.11 ± 12.80	39.60 ± 17.12	0.082	41.50 (10.00, 66.00)	33.00 (19.00, 61.00)	0.549
TPO (U/ml)	54.46 ± 72.57	86.02 ± 124.90	0.055	29.3 (5.7, 60.0)	99.9 (11.7, 533.1)	0.019*
TG (U/ml)	42.82 ± 54.46	91.83 ± 127.25	0.001*	34.05(10.11, 97.14)	82.10 (1.99, 500.00)	0.003*
T3 (nmol/l)	1.96 ± 1.60	4.01 ± 13.72	0.114	1.99 (0.66, 2.45)	1.20 (0.70, 88.08)	1.000
T4 (nmol/l)	97.47 ± 25.95	120.95 ± 65.17	0.002*	106.5 (76.9, 170.0)	94.8 (11.7, 363.6)	0.765
FT3 (pmol/l)	4.07 ± 0.97	4.45 ± 3.31	0.273	4.16 (2.59, 5.47)	3.89 (1.03, 23.98)	0.903
FT4 (pmol/l)	15.62 ± 4.67	20.21 ± 15.91	0.006*	17.06 (10.89, 22.92)	17.18 (8.60, 99.25)	0.663
TSH (uIU/ml)	1.91 ± 2.48	2.85 ± 6.05	0.169	1.93 (0.42, 4.02)	0.98 (0.01,36.50)	0.149

*Abbreviations: AHA* anti-histone antibody, *AKA* anti-keratin antibodies, *ANA* antinuclear antibodies, *ANCA* anti-neutrophil cytoplasmic antibodies, *ANuA* anti-nucleosome antibody, *C* complements, *CCP* anti-cytosolic carboxypeptidase antibodies, *CH50* 50% complement hemolysis, *CRP* C-reactive protein, *CSF* cerebrospinal fluid, *Ds-DNA* anti-double stranded DNA antibodies, *ESR* erythrocyte sedimentation rate, *FT3* free triiodothyronine, *FT4* free thyroxin, *IgG* immunoglobulin G, *Jo-1* anti-Jo-1 antibodies, *NMO-IgG* neuromyelitis optica immunoglobulin G, *NMOSD* neuromyelitis optica spectrum disorders, *OCB* oligoclonal banding, *RA33* anti-RA33 antibodies, *RF* rheumatoid factor, *Rib-P* anti-ribosomal P protein antibodies, *RNP* anti-ribonucleoprotein antibodies, *Scl-70* anti-topoisomerase I antibodies, *Sm* anti-Sm antibodies, *SSA* Anti-SSA/Ro antibodies, *SSB* anti-SSB/La antibodies, *T3* triiodothyronine, *T4* thyroxine, *TG* anti-thyroglobulin antibodies, *TSH* thyroid-stimulating hormone, *TPO* anti-thyroid peroxidase antibodies, *WBC* white blood cell.

**p < 0.001, *p < 0.05.

NMOSD patients with autoimmune diseases were divided two groups with non-organ-specific and organ-specific autoimmune diseases. There were no statistical differences in CSF indexes between two groups (p > 0.05). ANA, SSA, SSB seropositivity was significantly higher in NMOSD patients with non-organ-specific autoimmune diseases than in NMOSD patients with organ-specific autoimmune diseases (p < 0.05), while anti-thyroid peroxidase antibodies (TPO) and TG seropositivity was significantly lower in NMOSD patients with non-organ-specific autoimmune diseases than in NMOSD patients with organ-specific autoimmune diseases (p < 0.05). NMO-IgG was detectable in 81.8% (18/22) NMOSD with non-organ-specific autoimmune diseases, while in 77.8% (14/18) NMOSD patients with organ-specific autoimmune diseases (p = 0.751). No difference was found in other autoantibodies, complements, and thyroid hormones between two groups (p > 0.05).

**Figure 2:**
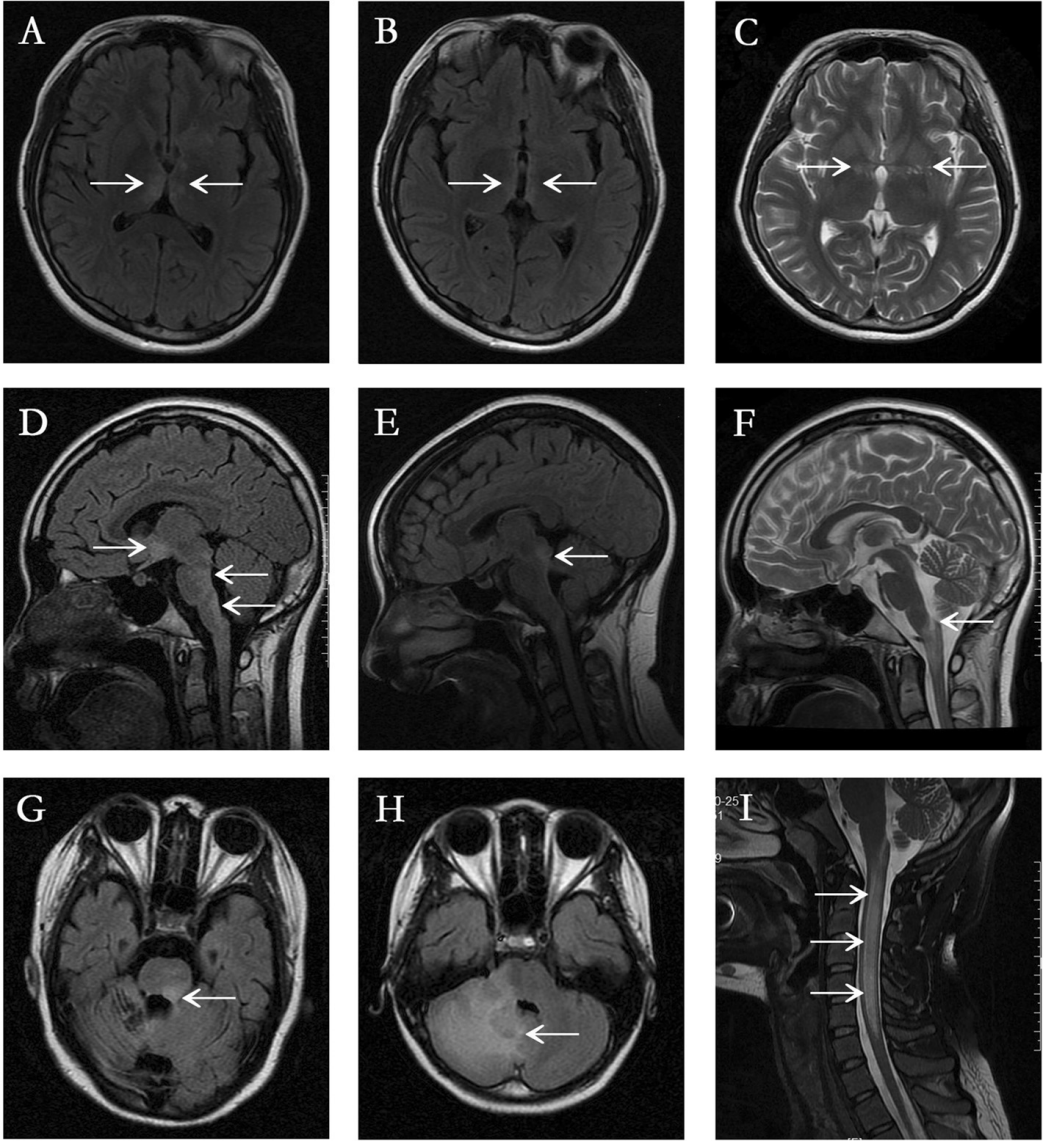
**Representative magnetic resonance imaging (MRI) abnormalities (arrows) in NMOSD patients with autoimmune diseases. (A)** Bilateral thalamic lesions; **(B)** Bilateral hypothalamus lesions; **(C)** Basal ganglia lesion; **(D)** Hypothalamus and periventricular lesions; **(E)** Aqueduct of the midbrain lesion; **(F)** Cervico-medullary lesion; **(G)** Pons lesion; **(H)** Cerebellum lesion; **(I)** Longitudinally extensive transverse myelitis (LETM) lesions.

As shown in Table 3 and Figure 2, NMOSD patients with autoimmune diseases had higher brain abnormalities than NMOSD without autoimmune diseases (100.0% vs 67.0%, p < 0.001). However, there was no statistical difference in other MRI features between two group divided methods (p > 0.05). Longitudinally extensive transverse myelitis (LETM) lesions on spinal cord MRI were noted in 70.4% (81/115) NMOSD patients without autoimmune diseases and in 70.0% (28/40) NMOSD patients with autoimmune diseases.

**Table 3:** MRI features between NMOSD without and with autoimmune diseases, and between NMOSD with non-organ-specific and organ-specific autoimmune diseases

	**NMOSD**			**NMOSD with autoimmune diseases**		
	**Without autoimmune diseases (n = 115)**	**With autoimmune diseases (n = 40)**	**P**	**Non-organ-specific (n = 22)**	**Organ-specific (n = 18)**	**P**
**Brain lesions, n (%)**	77 (67.0%)	40 (100.0%)	<0.001**	22 (100.0%)	18 (100.0%)	-
Brain lobes	50 (43.5%)	23 (57.5%)	0.126	12 (54.5%)	11 (61.1%)	0.676
Basal ganglia	15 (13.0%)	8 (20.0%)	0.286	6 (27.3%)	2 (11.1%)	0.382
Hypothalamic and thalamic	12 (10.4%)	2 (5.0%)	0.302	1 (4.5%)	1 (5.6%)	1.000
Ventricle and aqueduct	21 (18.3%)	10 (25.0%)	0.359	4 (18.2%)	6 (33.3%)	0.463
Midbrain	5 (4.2%)	2 (5.0%)	0.864	1 (4.5%)	1 (4.5%)	1.000
Pons	14 (12.2%)	9 (22.5%)	0.114	4 (18.2%)	5 (27.8%)	0.732
Medulla oblongata	29 (25.2%)	13 (32.5%)	0.372	5 (22.7%)	8 (44.4%)	0.145
Cerebellum	8 (7.0%)	1 (2.5%)	0.299	1 (4.5%)	0 (00.0%)	1.000
**Spinal cord lesions, n (%)**	88 (76.5%)	32 (80.0%)	0.650	17 (77.3%)	15 (83.3%)	0.634
Segments lesions	5 (0, 17)	6 (0, 15)	0.486	4 (0, 17)	6.5 (0, 15)	0.603
LETM	81 (70.4%)	28 (70.0%)	0.959	14 (63.6%)	14 (77.8%)	0.332
Cervical cord	74 (64.3%)	27 (67.5%)	0.719	12 (54.5%)	15 (83.3%)	0.053
Thoracic cord	62 (53.9%)	26 (65.0%)	0.223	14 (63.6%)	12 (66.7%)	0.842
Cervical and thoracic cord	48 (41.7%)	23 (57.5%)	0.085	11 (50.0%)	12 (66.7%)	0.289

*Abbreviations: LETM* longitudinally extensive transverse myelitis, *MRI* magnetic resonance imaging, *NMOSD* neuromyelitis optica spectrum disorders.

**p < 0.001.

## Discussion

In the present study, we found that NMOSD with autoimmune diseases have higher frequency of brain abnormalities than NMOSD without autoimmune diseases. We also found most clinical, laboratory, and MRI features do not differ significantly between different NMOSD subtypes divided by autoimmune diseases, while several CSF indexes, autoantibodies, and thyroid indexes differ significantly.

Motor deficit is typical feature of NMOSD. Furthermore, we found motor deficit was less frequency in patients with non-organ-specific autoimmune diseases. That finding was consistent with our spinal cord MRI findings that cervical cord lesions were less prevalent in patients with non-organ-specific autoimmune diseases, though the difference was not statistically significant. Intractable hiccup and nausea (IHN) is rare and unique symptom in NMOSD. Several studies have reported IHN can be seen in 15.7-62.0% NMO patients [11],[12]. In our case series, 18.2-44.4% patients had IHN, though the difference was not statistically significant. Area postrema and nucleus tractus solitarius in the dorsomedial medulla and ventrolateral respiratory center involvement were the reasons for IHN. Previous report had found dorsal and pericanal regions of the medulla oblongata are frequently involved in NMO [13]. One of the explanations for this regional preference is the relative abundance of aquaporin-4 (AQP4) expression and compromised blood–brain barrier functions in these areas. Though clinical observations strongly suggested that many patients with NMO have neuropathic pain (NP), few systemic studies have focused on NP in NMOSD. Elsone et al. reported 27.3% of NMO showed neuropathic pruritus, a special NP [14]. However, we describe the cases of 25.4% and 32.5% with NP in our patients. Kanamori et al. had found that pain in NMO is more frequent and severe than in multiple sclerosis [15]. NP may be related to the location of lesions in dorsal horn of spinal cord or spinal nucleus of trigeminal nerve or peri-aqueductal pathways [14]. Demographic and clinical features do not differ significantly between NMOSD without and with autoimmune diseases in our study.

In the current study, CSF WBC and protein, serum CRP and IgG were higher in NMOSD patients with autoimmune diseases. It suggests that the immune status is more active in patients with autoimmune diseases than that in patients without autoimmune diseases. Athough all NMOSD subtypes can found autoantibodies in the serum (especially ANA, SSA, SSB and RF in our study), different subtypes should have different autoantibodies seropositivity. These can be partly attributable to our inclusion criteria to recruit patients with different autoimmune diseases. In fact, ANAs are common in patients with NMOSD, especially in NMOSD with SLE/SS [4]. NMO patients show concomitant autoantibodies, variably from 38 to 75%, of which SSA is frequently detectable in NMO-IgG seropositive patients [4],[16]. The finding was in agreement with our present study. In one study, autoantibody markers of SS or SLE were found in 47% patients with NMOSD [17]. In addition, recurrent myelitis and NMO show positivity for SSA, more frequently (77%) than in monophasic disease (33%) [18]. NMO can be associated with autoimmune thyroid diseases, including chronic thyroiditis, Graves’ disease (GD) or benign thyroid tumours [19]. In the present study, all organ-specific autoimmune diseases patients were autoimmune thyroid diseases that might explain why TPO and TG seropositivity was frequent in that group. Previous study based on a large Japanese cohort reported autoimmune thyroid diseases were seen in 13.6% NMOSD and were more often observed in the brain-dominant phenotype (40.0%) [5]. The serum autoantibody NMO-IgG is a sensitive and specific marker for NMO. Its antigen is AQP4, the predominant water channel protein in the central nervous system [20]. Lennon and co-workers reported the presence of NMO-IgG was 91-100% seropositivity for NMO patients [20]. The autoantibody NMO-IgG is detectable also in patients with recurrent optic neuritis without myelitis and in a high proportion of patients with a single episode or recurrence of LETM without optic neuritis (NMOSD) [17]. NMO-IgG was detectable in 74-85% samples from patients with NMOSD [21],[22]. In our case series, 67.0-81.8% patients had NMO-IgG seropositivity, though the difference was not statistically significant. The reason why NMO-IgG positive in NMO/LETM frequently associated with autoimmune diseases is unclear. It has been speculated that the coexistence of the two disorders in the same patient might reflect a general autoimmune predisposition [23].

The frequency of brain MRI abnormalities is 50–85% in patients with NMO/NMOSD [24]-[28]. However, previous small study had reported all SS patients with NMOSD had several common brain abnormalities [29]. In present study, we found brain abnormalities in NMOSD with autoimmune diseases were more frequent than that in other NMOSD phenotypes. Among the brain lesions, asymptomatic lesions are more common; however, symptomatic brain involvement is also common [30]. Diencephalic or brainstem lesions adjacent to the third and fourth ventricles, longitudinal lesions of the internal capsule, and large extensive lesions have been suggested as characteristic lesions in NMO, even though they are not commonly observed [13],[24],[31]. Among the brain lesions, nonspecific lesions are very commonly found. They can be dot-like or patchy, < 3 cm in diameter, and located in the cerebral deep white matter, brainstem, or cerebellum [30]. The distribution of NMO-characteristic brain lesions corresponded to sites of high AQP4 expression, adjacent to the ventricular system at any level [13]. However, other NMO-characteristic brain lesions involved where AQP4 expression is not particularly high have also been reported [27],[30]. In our case series, patients had a higher frequency of medulla oblongata lesions than that of midbrain and pons lesions. The result was consistent with our previous report showing that the medulla was the most common brainstem lesion location in NMO [32]. MRI of the spine cord has been regarded as the most useful diagnostic test in patients with suspected NMO [33]. The combination of NMO-IgG and a longitudinally extensive (three or more vertebral segments) cord lesion has been shown to be highly specific for the diagnosis [2]. Lesions are typically located in the cervical or thoracic cord with central or holocord involvement [34]. In our case series, although the difference was not statistically significant between groups, over 60% NMOSD patients had LETM lesions on spine cord MRI.

Our study has some limitations. First, the number of NMOSD patients with autoimmune diseases included in our study is not sufficient. Second, the titer of NMO-IgG and autoantibodies was not tested, which result could strengthen our findings. Third, as a retrospective study, bias is inevitable. Furthermore, because the patients came from a single center, we were not able to validate our findings by applying them to a set of patients from other hospital. The p-values we reported were not corrected for multiple hypothesis testing. After post hoc analyses with Benjamini-Hochberg method, there were no statistical differences in CSF protein and glucose, AnuA and Sm seropositivity between NMOSD without and with autoimmune diseases. No difference was found in motor deficit and TPO between NMOSD with non-organ-specific and organ-specific autoimmune diseases.

## Conclusions

In conclusion, the clinical, laboratory, and MRI features were similar between different NMOSD subtypes. NMOSD with autoimmune diseases have high frequency of brain abnormalities.

## Competing interest

The authors declare that there are no conflicts of interest.

## Authors’ contributions

Study design: all authors. Data collection: BJZ, YZ, YQW. Statistical analysis: BJZ, YZ. Manuscript preparation: all authors. All authors read and approved the final manuscript.
